# Data-driven vs. psychological personality temperaments: theoretical and clinical utility of personality measures in psychiatry

**DOI:** 10.3389/fpsyt.2024.1436121

**Published:** 2024-12-05

**Authors:** Abdelrahman S. Sawalma, Mahmud A. Sehwail, Jürgen Dammers, Mohammad M. Herzallah

**Affiliations:** ^1^ Palestinian Neuroscience Initiative, Al-Quds University, Jerusalem, Palestine; ^2^ Institute of Neuroscience and Medicine (INM-4), Forschungszentrum Jülich, Jülich, Germany; ^3^ Faculty of Medicine, Rheinisch-Westfälische Technische Hochschule (RWTH) Aachen University, Aachen, Germany; ^4^ Center for Molecular and Behavioral Neuroscience, Rutgers University, Newark, NJ, United States

**Keywords:** personality, temperaments, dimension, temperament and character theory, tri-dimensional personality questionnaire, independent component analysis, major depressive disorder

## Abstract

Decades of research on personality identified dissociable psychological temperaments. Cloninger’s temperament and character theory used a psychobiological approach to differentiate three major dimensions of personality: harm avoidance, novelty seeking, and reward dependence. Previous studies, heretofore, did not examine the correspondence between Cloninger’s psychological temperaments and statistically independent data-driven components and how that could enhance the clinical utility of personality temperaments. In this study, we validated an Arabic version of the tri-dimensional personality questionnaire (TPQ) to construct data-driven personality temperaments using independent component analysis (ICA). Using SVM, we contrasted the clinical utility of data-driven personality vs. Cloninger’s psychological temperaments in differentiating medication-naïve patients with major depressive disorder (N=244) and healthy subjects (N=1109). Data-driven personality components based on ICA showed very little overlap with Cloninger’s original temperaments. Both Cloninger’s temperaments and data-driven components revealed low internal consistency (for subscales) but high test-retest reliability. Cloninger’s temperaments, however, showed a poor goodness-of-fit for the structure of the TPQ. Data-driven components significantly outperformed psychological TPQ temperaments with higher accuracy and recall but not precision. To our knowledge, this is the first study to examine the clinical utility of data-driven vs. psychological personality metrics using a sizeable sample of patients and healthy individuals. Our results could have wide implications for reexamining psychometric data to extract data-driven latent structures that can improve replicability, clinical utility, and cross-disciplinary inference.

## Introduction

A myriad of psychological models explored the principal dimensions of personality. Collectively, these models varied in the number of proposed psychological dimensions of personality based on different survey methods, including the three-factor model (1, 2), the big-five model (3, 4), Strelau’s temperament inventory (5), Catell’s 16 personality factor model (6), the alternative model of personality disorders (AMPD) (7), and the Norman five (8). In the past three decades, Cloninger’s psychobiological model of personality (9, 10) inferred significant impact on the field of psychiatry (11). Cloninger’s model is based on neurochemical, neuroanatomical, and neurogenetic evidence, especially linking to monoaminergic activity in the brain. Thus, Cloninger’s model presents a more suitable tool to study personality temperaments across psychiatric disorders (9, 10).

According to Cloninger, personality is expressed in three temperaments: novelty seeking (NS), representing exploration and impulsive actions; harm avoidance (HA), defined as cautiousness and fear of negative outcomes; and reward dependence (RD), manifested as seeking and maintenance of actions that result in positive outcomes (10). Each of these temperaments is further divided into four subscales according to different sub-temperaments. Cloninger used this categorization to create the tri-dimensional personality questionnaire (TPQ; 100 true/false questions) (10). Both English and translated versions of the TPQ revealed uniformly acceptable internal consistency and cross-cultural similarity [English (12), French (13), Japanese (14) and German (15, 16)]. It remains elusive, however, to what extent the data structure of question/item responses in the TPQ conform to the different temperaments put forward by Cloninger.

Previous studies utilized exploratory factor analysis (EFA) to examine the uncorrelated data components based on subscales and verify their correspondence with Cloninger’s personality temperaments in the TPQ (15–19). Most studies reported results that are largely consistent with Cloninger’s model (15, 16). Nevertheless, factors loadings were not unique to one subscale only, and some of them exhibited considerable cross-loadings on more than one subscale. Similarly, confirmatory factor analysis (CFA) results were inconclusive to support personality temperaments proposed by Cloninger (19–21). This is further corroborated by internal consistency measures (Cronbach’s alpha) to test the structure of TPQ. At the temperament level, measures of internal consistency were within acceptable range (12). However, many of the subscales showed low internal consistency values (12, 19). Taken together, these lines of evidence show that the TPQ temperament and sub-temperament structure is not fully supported by data-driven constructs.

Various proposals appeared to restructure TPQ based on its questions/items using EFA (14, 22–24). Collectively, these studies failed to replicate Cloninger’s structure and their internal consistency remained low. Moreover, EFA comes with multiple limitations related to the assumption of normality (Gaussianity) (25, 26) which cannot be expected in the TPQ where the answers are dichotomous (true or false). Further, in case of non-Gaussian data, EFA will only result in decorrelation of the signal and not statistically-independent components (27). Independent component analysis (ICA), on the other hand, could decompose data into statistically independent components with no prior information about the nature of the underlying sources (28, 29). Statistical independence in ICA is computed using higher order moments, which are stronger statistical properties when compared to decorrelation (30). This warrants the use of ICA to identify the independent, rather than the uncorrelated, components when trying to find the personality components underlying the TPQ.

Personality components and temperaments were heavily studied in relation to psychiatric disorders (31). Compared to other models of personality, Cloninger’s model in the TPQ showed significant promise in quantifying personality changes in psychiatric disorders. For instance, the TPQ showed higher HA in generalized anxiety disorder (32), panic disorder (32), eating disorders (33), obsessive compulsive disorder (34), following exposure to psychological trauma (35), and as a predictor of development of post-traumatic stress disorder (PTSD) (36). Conversely, patients with PTSD, alcoholism, and substance abuse exhibited higher levels of NS (16, 37). The correlation of TPQ temperaments and major depressive disorder (MDD) has received special attention. TPQ is characterized by high HA scores in patients with MDD (38, 39), as well as in healthy siblings of patients with MDD (40). Furthermore, TPQ temperaments, especially HA, were predictive of response to treatment in MDD (41, 42). However, significant overlaps are present within psychiatric disorders and within personality dimensions, which makes it difficult to discern independent personality dimensions when studying the clinical utility of TPQ, as evident in previous studies.

In general, a critical gap in studies of psychiatric disorders lies in the dissociation between the effects of these disorders and those stemming from exposure to psychotropic medications. The same applies to previous studies of TPQ in psychiatric populations. Previous studies recruited and mixed patients on-medications, patients off-medications, and medication-naive patients. The lack of dissociation between disorder and medication effects could introduce multiple confounding variables (43, 44). Effects of chronicity (45) combined with the immediate vs. long-term effects of treatment (46) can also mask the personality dimensions of psychiatric disorder. This is further compounded by the vast comorbidity between different psychiatric disorders (47).

In this study, we used ICA to analyze TPQ at the question/item level to explore independent components based on a data-driven personality construct. We illustrated the differences between the data-driven and psychological personality temperaments as proposed by Cloninger. Further, we assessed the clinical utility of data-driven vs. psychologically-defined personality temperaments in patients with MDD. In particular, we attempted to circumvent previous limitations of studies on psychiatric populations by testing medication-naïve patients with MDD. For all aforementioned analyses, we used EFA to analyze TPQ results as a control condition. Compared to Cloninger’s temperaments and EFA results, we predicted that our data-driven temperaments will be principally different and possess diagnostic and treatment-predictive utilities that outperform the psychological temperaments.

## Methods

### Protocols and consent

All subjects provided written informed consent before enrollment in the study. Study protocols were in accordance with the Declaration of Helsinki and approved the Al-Quds University Research Ethics Committee.

### Subjects

We tested 1353 Arabic-speaking subjects in the West Bank, Palestine, including 244 patients with MDD and 1109 healthy controls (HC). A subsample of 90 HC was tested and retested 4-6 weeks apart to check for the test-retest reliability of the questionnaire. Participants were recruited from Al-Quds University, An-Najah University, and various psychiatric and neurological clinics throughout the West Bank, Palestine.

Exclusion criteria for all subjects included prior psychotropic drug exposure; the presence of any psychiatric disorder except for MDD, major medical or neurological illness, illicit drug use or alcohol abuse within the past year, lifetime history of alcohol or drug dependence and current pregnancy or breastfeeding.

Aside from completing an Arabic-translated version of the TPQ (48), all subjects were interviewed using the mini-international neuropsychiatric interview (MINI) (49), the Beck depression inventory II (BDI-II) (50) and the Beck anxiety inventory (BAI) (51). **Table 1** summarizes demographics and neuropsychological characteristics for our sample.

**Table 1:** Demographics and basic psychometric measures of subjects in both the HC and MDD groups. Age and Education are given in years and are presented as mean ± standard deviation.

	HC	MDD
**Sample Size**	1109	244
**Age**	22.3 ± 9.2	28.6 ± 9.7
**Education**	14.0 ± 3.6	12.7 ± 3.0
**Female Percentage**	59.6	60.7
**BDI score**	10.1	30.7
**BAI score**	10.6	28.3

### TPQ validation

For initial validation of the translation, the TPQ was translated to Arabic and back-translated to English for cross-referencing. Following data collection, we used HC data for validation of our Arabic translation of the TPQ. In particular, we calculated Cronbach’s alpha for internal consistency estimation for TPQ scales and subscales. Further, we performed a test-retest reliability on a subset of 90 subjects using Spearman’s rho. Internal consistency measures for the main scales were within the acceptable range for the three main scales, ranging from 0.63 to 0.82. Cronbach alpha scores were acceptable for HA subscales and poor for NS and RD subscales, except for RD3, which had acceptable score of 0.70. Test-retest reliability was moderately high for the main scales, ranging from 0.70 to 0.81, and moderate to high in the subscales, ranging from 0.42 to 0.8. **Table 2** shows the descriptive statistics for TPQ scales and subscales along with Cronbach alpha values. Our validation results were similar to previous validation and normative studies of the TPQ (12, 15, 16, 19).

**Table 2:** Descriptive statistics and internal consistency measures for HC, divided into males and females.

TPQ Measure	Mean All (mean ± SD)	Mean Male (mean ± SD)	Mean Female (mean ± SD)	Cron. α All	Cron. α Male	Cron. α Female	Test-retest Reliability
**NS1**	5.1 ± 1.6	5.0 ± 1.6	5.2 ± 1.6	0.35	0.35	0.35	0.57*
**NS2**	2.6 ± 1.7	2.6 ± 1.6	2.5 ± 1.8	0.50	0.45	0.54	0.58*
**NS3**	3.5 ± 1.6	3.5 ± 1.6	3.5 ± 1.6	0.55	0.53	0.58	0.81*
**NS4**	4.6 ± 1.9	4.6 ± 1.9	4.5 ± 1.8	0.39	0.42	0.36	0.73*
**NS**	15.7 ± 4.2	15.8 ± 4.1	15.8 ± 4.3	0.63	0.60	0.65	0.70*
**HA1**	3.5 ± 2.1	3.5 ± 2.1	3.5 ± 2.2	0.61	0.57	0.64	0.66*
**HA2**	4.0 ± 1.9	4.0 ± 1.9	4.0 ± 1.9	0.63	0.65	0.63	0.67*
**HA3**	1.9 ± 1.6	1.9 ± 1.7	1.9 ± 1.7	0.60	0.60	0.60	0.80*
**HA4**	3.6 ± 2.5	3.5 ± 2.5	3.7 ± 2.6	0.73	0.72	0.74	0.78*
**HA**	13.0 ± 5.9	12.8 ± 5.8	13.1 ± 6.1	0.82	0.81	0.83	0.81*
**RD1**	4.3 ± 0.9	4.3 ± 1.0	4.4 ± 0.9	0.41	0.45	0.40	0.56*
**RD2**	4.1 ± 1.7	4.3 ± 1.6	4.0 ± 1.8	0.38	0.29	0.43	0.42*
**RD3**	7.0 ± 2.5	6.8 ± 2.4	7.1 ± 2.5	0.70	0.68	0.72	0.72*
**RD4**	2.0 ± 1.3	1.9 ± 1.3	2.0 ± 1.3	0.45	0.41	0.47	0.63*
**RD**	17.4 ± 3.8	17.2 ± 3.7	17.5 ± 3.9	0.61	0.58	0.62	0.71*

SD, standard deviation *: significant Spearman’s rho at p < 0.001.

### Exploratory & confirmatory factor analysis for TPQ subscales

We performed exploratory factor analysis (EFA) on the 12 scales of the TPQ using a three-factor solution with a varimax rotation (52). We followed EFA with confirmatory factor analysis (CFA) using the *lavaan* R package to measure the resemblance between the resulting factors and the original Cloninger scales (53). CFA included measures of goodness of fit: p-value, comparative fit index (CFI), root mean square error of approximation (RMSEA) and the standardized root mean square residual (SRMR). We used the following criteria to for goodness of fit: a non-significant chi-square p-value (20), CFI > 0.90 (54), RMSEA < 0.08 (54) and SRMR <0.10 (54).

### Independent component analysis

**Figure 1:**
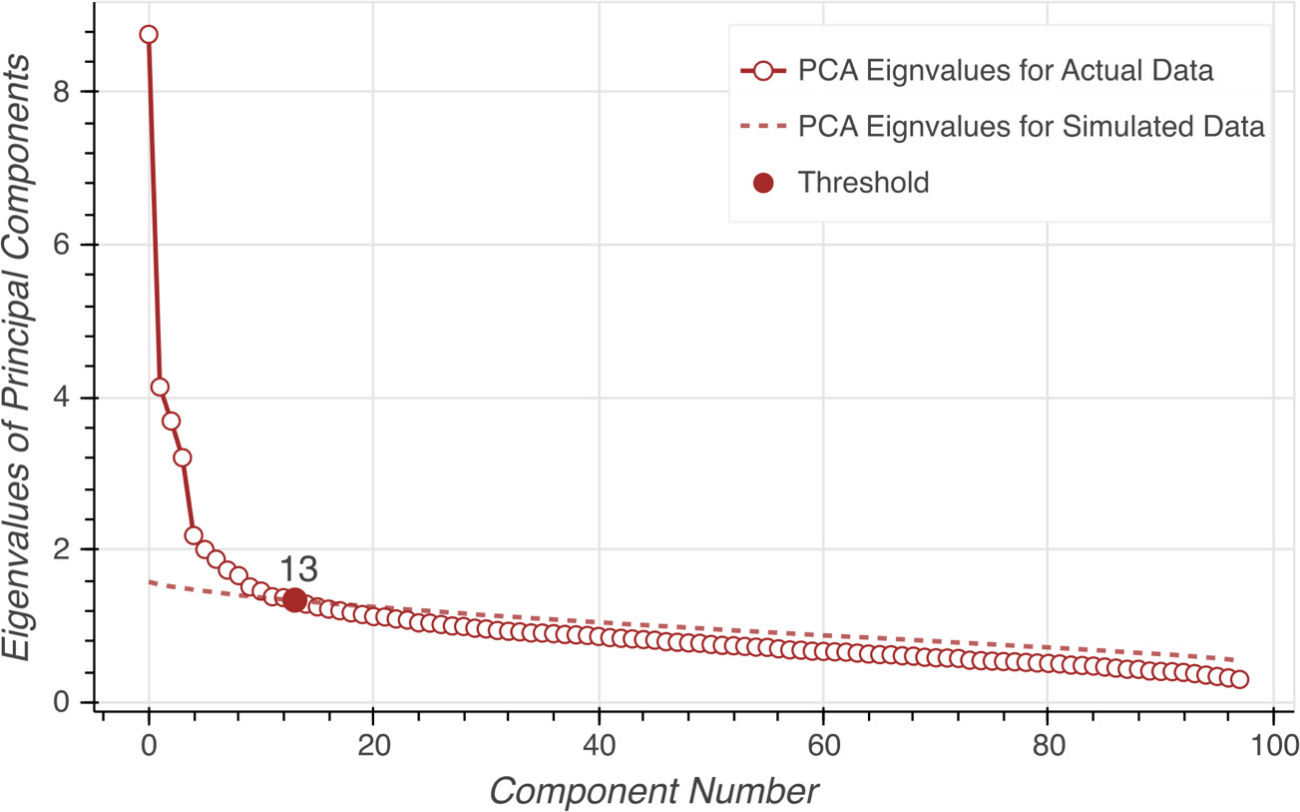
Parallel analysis on eigenvalues to estimate the number of source components. The dashed line represents eigenvalues for the simulated data. The circles represent eigenvalues for the actual data. The red dot indicates the estimated number of components.

We applied ICA at the item/question level to extract independent components of personality in our volunteers. In particular, we used Infomax ICA which utilizes an unsupervised neural network for maximizing the joint entropy to find the independent components (28, 55, 56). Prior to applying ICA, principal component analysis (PCA) was applied for data whitening and dimension reduction (26). Using parallel analysis, we were able to determine the appropriate number of PCA components to retain post whitening. In parallel analysis, we created a simulated dataset from which we calculated the eigenvalues. The number of components is determined as the number of original data components with eigenvalues higher than the intersection point between simulated data components (57). In our dataset, using parallel analysis indicated that the number of components that pass the parallel analysis threshold in our dataset was 13 (from 100). We wrote the code for parallel analysis in Python based on the R package *psych* (58). Parallel analysis results are illustrated in **Figure 1**. Accordingly, our subsequent ICA was expected to produce 13 independent components (ICs) based on the TPQ data.

### Support vector machine

We used SVM to compare the ability of ICA data-driven components vs. Cloninger’s original TPQ temperaments in differentiating patients with MDD from HC. The ICA dataset contained 13 independent components extracted from TPQ questions/items using Infomax ICA. The temperament subscale dataset separated TPQ results into 12 subscales according to the original approach suggested by Cloninger (10).

Our sample contained 1109 HC and 244 patients with MDD. We split the dataset using an 80/20 ratio for training and holdout. In order to overcome the limitations of the imbalanced dataset, we used a combination of undersampling of the majority group, and oversampling the minority group. For oversampling, we utilized synthetic minority oversampling technique (SMOTE) (59) which introduces new examples in the minority class along the line segments joining the k nearest neighbors (59, 60). SMOTE was combined with undersampling, which is done by randomly removing samples from the majority group as implemented in the python package *imbalanced-learn* (61). Studies suggest that using SMOTE in combination with random undersampling provides better classification results as compared to undersampling only (59). We applied he SMOTE/undersampling approach to the training and holdout datasets independently.

**Figure 2:**
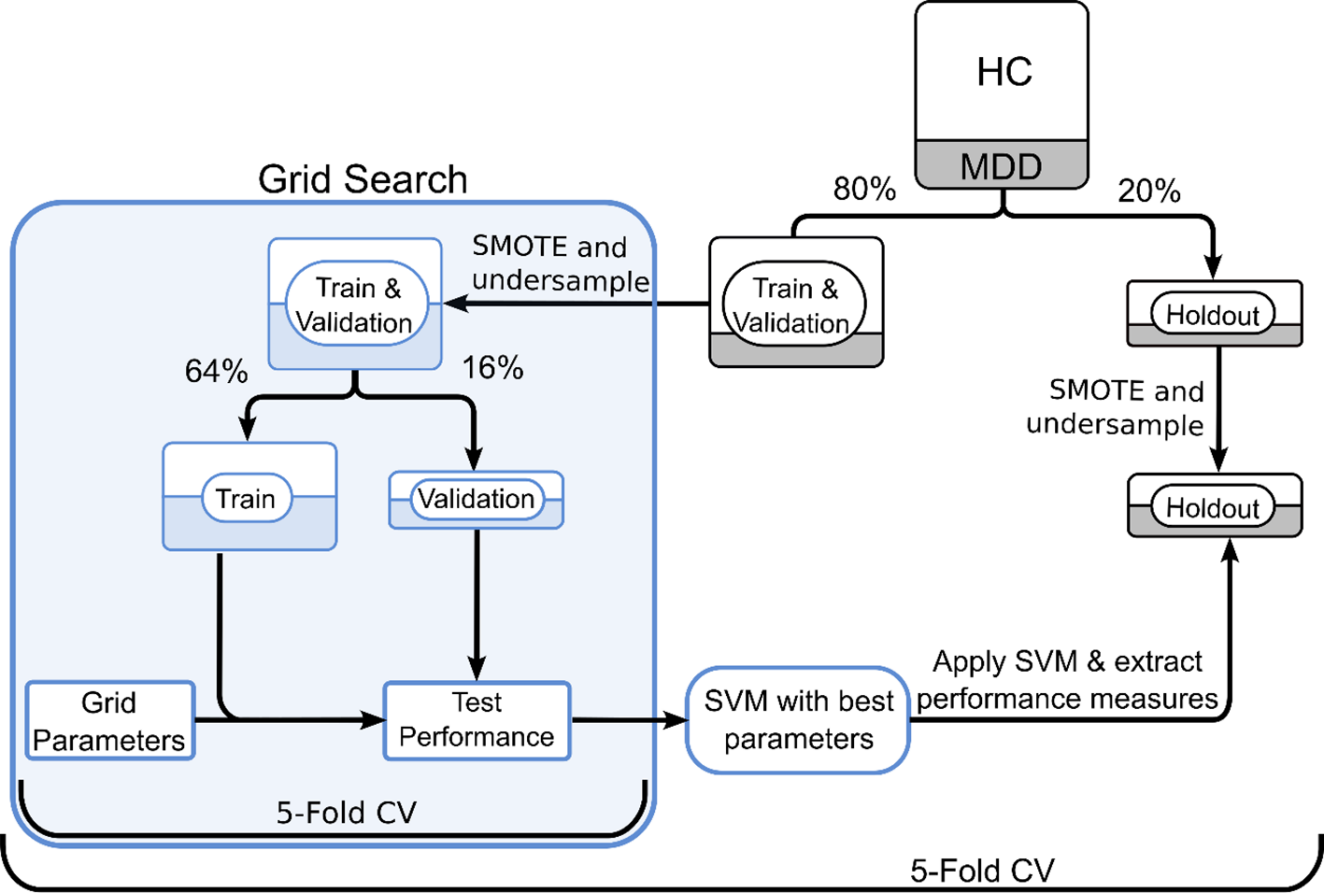
SVM workflow for subscales and ICA datasets. Grid search was used in a 5-fold cross validation paradigm to finetune hyperparameters. The entire SVM workflow followed 5-fold cross-validation to avoid potential undersampling bias. CV, cross validation.

We applied SVM on the balanced training dataset to test the clinical utility of ICA data-driven vs. subscale variables vs TPQ factors in identifying patients with MDD (62). The data were divided into training, validation and holdout datasets with ratios 64/16/20. Optimal hyperparameters were estimated using grid search as implemented in Python *scikit-learn (* 63). Grid search compares combinations of different types of kernels (e.g., linear, radial basis function (RBF) or sigmoid) and kernel function parameters (hyperplane confidence interval (C), linearity of separation (γ)) to find the optimal hyperparameters. The median hyperparameters are shown in **Table 3**. To prevent overfitting, SVM was run 5 times in a 5-fold cross validation scheme, where we changed the subjects assigned to the holdout set with every iteration. A workflow of the model training and testing is shown in **Figure 2**.

**Table 3:** Best parameters chosen by grid search cross validation for each data set.

Hyperparameter	Subscales Dataset	ICA Dataset	FA Dataset
C	5	3	3
γ	0.1	0.1	25
Kernel	RBF	RBF	RBF

Grid search cross validation was applied 4 times, one for each training data set produced. The median of the parameters used are mentioned here. RBF, radial basis function.

## Results

### EFA and CFA of TPQ

We used EFA to investigate whether uncorrelated factors of TPQ subscales conform to Cloninger’s TPQ scales. Factor loadings of subscales following varimax rotation are shown in **Table 4**. For simplification, we removed all absolute factor loadings below 0.3. The three-factor solution largely replicates Cloninger’s three temperaments, with some deviations in NS1, HA2 and RD2. The variance explained by the factor model was 33%.

**Table 4:** Factor loadings onto TPQ subscales for a three-factor solution using EFA.

	F1	F2	F3
**NS1**	-0.37		
**NS2**		0.49	
**NS3**		0.34	
**NS4**		0.64	
**HA1**	0.74		
**HA2**	0.56	-0.34	
**HA3**	0.67		
**HA4**	0.7		
**RD1**			-0.35
**RD2**			
**RD3**			-0.68
**RD4**			-0.43
**Cumulative Variance**	0.17	0.26	0.33

Shown here are loadings with absolute value > 0.3.

We used CFA to confirm whether Cloninger’s three temperament model of TPQ fits our data. CFA measures showed a poor goodness-of-fit. Three measures showed poor goodness-of-fit while the fourth was acceptable. CFA goodness-of-fit results can be found in **Table 5**.

**Table 5:** Goodness-of-fit values for the CFA Model.

Measure	Value
χ^2^	460.936
p-value	<0.001
CFI	0.744
RMSEA	0.085
SRMR	0.083

CFI, comparative fit index; RMSEA, root mean square error of approximation; SRMR, standardized root mean square residual.

### Data-driven components of the TPQ using ICA

To complement our analysis on the original TPQ temperaments and subscales, we conducted both Cronbach’s alpha and a test-retest reliability using Spearman’s rho on the data-drive personality components. ICA produced 13 components that represent the data-driven TPQ structure. In order to project the components onto the question space, we assigned each component with the questions with the highest 10th percentile of loadings. Below is a summary of the internal consistency and test-retest reliability of the data-driven components (**Table 6**).

**Table 6:** Descriptive statistics and internal consistency measures for the derived independent components.

	Mean All (mean ± SD)	Mean Male (mean ± SD)	Mean Female (mean ± SD)	Cron. α	Cron. α Male	Cron. α Female	Test-Retest Reliability
IC1	4.0 ± 2.5	4.1 ± 2.5	3.9 ± 2.5	0.71	0.72	0.71	0.619
IC2	5.2 ± 2.4	5.1 ± 2.3	5.3 ± 2.4	0.66	0.63	0.68	0.696
IC3	2.9 ± 1.8	3.0 ± 1.7	2.9 ± 1.8	0.41	0.32	0.47	0.606
IC4	4.8 ± 1.7	4.7 ± 1.6	5.0 ± 1.6	0.22	0.2	0.22	0.736
IC5	4.2 ± 2.0	4.3 ± 1.9	4.2 ± 2.1	0.46	0.39	0.51	0.583
IC6	5.6 ± 1.6	5.5 ± 1.6	5.7 ± 1.6	0.13	0.1	0.15	0.747
IC7	4.6 ± 2.3	4.4 ± 2.2	4.8 ± 2.4	0.65	0.61	0.67	0.623
IC8	4.4 ± 1.7	4.5 ± 1.8	4.2 ± 1.7	0.37	0.39	0.36	0.825
IC9	4.7 ± 1.7	4.5 ± 1.7	4.8 ± 1.7	0.29	0.25	0.32	0.654
IC10	4.5 ± 1.6	4.6 ± 1.7	4.5 ± 1.6	0.17	0.24	0.09	0.644
IC11	4.3 ± 1.7	4.3 ± 1.6	4.3 ± 1.7	0.2	0.14	0.25	0.579
IC12	4.8 ± 2.0	4.6 ± 2.0	5.0 ± 2.0	0.47	0.43	0.49	0.676
IC13	3.9 ± 1.7	3.9 ± 1.7	3.8 ± 1.7	0.2	0.19	0.21	0.503

Each component is represented by the 10 questions that have the highest weight. SD, standard deviation; *, significant Spearman’s rho at p < 0.001.

**Figure 3:**
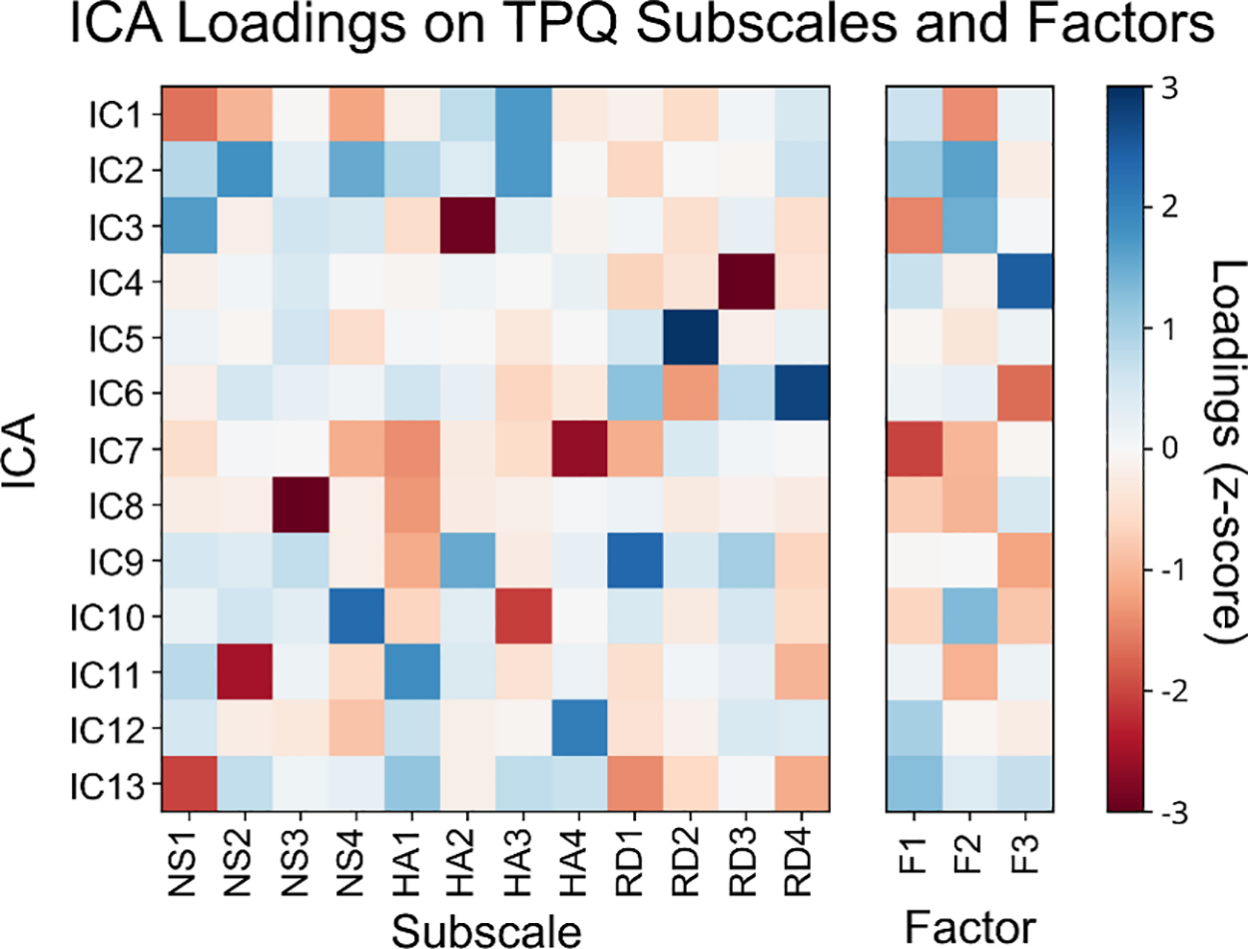
ICA loadings on TPQ subscales (left) and TPQ factors (right).

In order to understand whether a correlation between the ICs and the TPQ subscales and factors, we identified the loading of the separate ICs on the Subscales and factors. For the subscales, we summed the ICA weights for the questions that represent each subscale. As for the TPQ factors, we summed the product of IC weights and factor weights for each IC and factor. The z-scored ICA loadings are represented in **Figure 3**.

### Identifying MDD using TPQ psychological vs. data-driven temperaments

**Figure 4:**
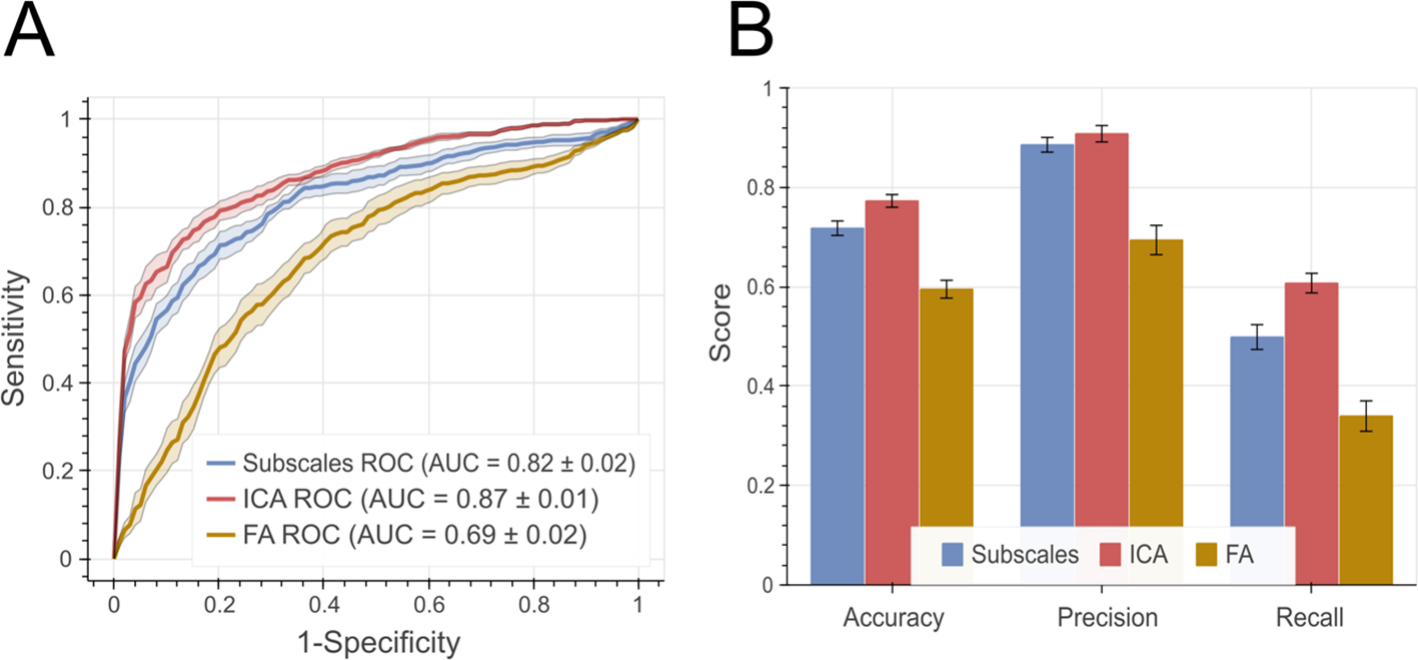
Performance measures for SVM-based classification. **(A)** ROC curves for both models with area under the curve (AUC) summaries at the bottom. The shaded area represents standard error of the mean. **(B)** shows the balanced accuracy, precision and recall measures. Error bars represent standard error of the mean. ROC, Receiver operating characteristic; AUC, Area under the curve.

We used the SVM-based classification workflow to compare data-driven ICA components to TPQ subscales and TPQ Factors for identifying MDD. Kruskal-Wallis Test was conducted to examine the differences in accuracy, precision, recall and AUC. The results show a significant difference in all four comparisons (Kruskal-Wallis H = 11.1, 9.78, 11.81 and 11.58, respectively, p-value <0.008 for all four comparisons). To follow up on this test, we performed Mann-Whitney’s test for the four comparisons with Holm-Bonferroni correction. For accuracy, recall and AUC comparisons, ICA-based SVM was higher than both the subscales- and the FA-based SVM, with corrected p-values <= 0.035 in all cases. As for precision, ICA-based SVM was not significantly different from Subscales-based SVM, and both were higher than FA-based SVM, with corrected p-values = 0.024 in both cases. Thus, our SVM analysis on ICA components outperformed the one based on Cloninger’s TPQ subscales in accuracy, recall and area under the curve (AUC), while precision was comparable, as summarized in **Figure 4**.

## Discussion

We investigated the data-driven vs. psychological structure of an Arabic-translation of Cloninger’s TPQ and its clinical utility. Our results revealed a discordance between data-driven and theory-driven classification of TPQ data. TPQ internal consistency and test-retest reliability results echoed previous findings by showing acceptable levels for the main three temperaments, but subscales did not perform as well. Using EFA produced a three-factor solution with loadings close to Cloninger’s subscale proposal. However, CFA exhibited a poor goodness-of-fit for the three-factor solution. Data-driven personality components using ICA did not corroborate Cloninger’s classification. Similar to Cloninger’s subscales, data-driven personality components showed low internal consistency but high test-retest reliability. In its clinical utility, data-driven personality components showed significantly better accuracy and recall for identifying patients with MDD than Cloninger’s theory-driven personality temperaments.

This study utilized Cloninger’s model given its psychobiological basis, especially the link between personality temperaments to various monoamines (9, 10). In contrast, other models of personality, such as the big five and its derivatives, assume that personality differences are lexically reflected in language (4, 64). Thus, their utility would better fit socio-cultural rather than psychobiological studies (65). The structure of temperament questionnaire (STQ) covers behaviorally-based components without sufficient inference to basic biological processes (66). More recent developments such as the functional ensemble of temperament (FET) were conceived after the initiation of data collection for the current study (67). Other clinically-oriented models, like the AMPD (included in the diagnostic and statistical manual of mental disorders (DSM-5)) (7), provides clinical evaluation of personality disorders (pathological personality), as opposed to personality temperaments in psychiatric disorders (68). With neurochemical, neuroanatomical, and neurogenetic basis, Cloninger’s model presented the most befitting choice to investigate personality temperaments in MDD (9, 10).

Our internal consistency results corroborated previously published normative data in English and translated versions of the TPQ (12, 15, 16, 19). Specifically, major temperaments showed high (for HA) or acceptable (NS and RD) internal consistency. In contrast, internal consistency was acceptable for HA subscales but low for NS and RD subscales. Our data show similar results for HA main scale and subscales, but lower internal consistency for RD and NS scales and subscales, falling below 0.60 in most subscales. Test-retest reliability was moderately high, confirming the reliability of TPQ when applied at different time points, which falls in line with previous findings as well (10, 12). Some of the inconsistencies with previous findings could be attributed to our significantly larger sample size. Further, we used stricter inclusion criteria for HC which could have unraveled previously unobserved characteristics of TPQ metrics.

We tested whether Cloninger’s three-temperaments of the TPQ using both EFA and CFA. Overall, EFA produced a three-factor solution close to that proposed by Cloninger with some deviations. In line with previous studies, the HA2 subscale showed high loading on the EFA factor for NS (16, 19) while RD2 did not have loadings on any of the three EFA factors (18). For validation of the goodness-of-fit of the three-factor solution of TPQ, we utilized CFA to test the extent to which the data fits the proposed variable structure (20). We applied CFA on the subscale level to test if the main scales can be reliably derived from the subscales. Our results revealed a poor goodness of fit for Cloninger’s model, consistent with previous findings (19, 69). Surprisingly, previous examination of CFA results on the TPQ overlooked the poor goodness-of-fit results and interpreted other metrics, such as the goodness of fit and root mean square, in isolation (20, 21). Taken together, our EFA and CFA results and previous findings indicated that there is more to the data-driven structure of the TPQ than what Cloninger’s model provides.

With ICA, we utilized more objective, non-Gaussian, and assumption-free approach to unravel the data-driven structure of TPQ. Previous methods to describe the structure of the TPQ were either subjective, i.e., the researcher deciding which questions belong to which scale based on *a priori* theoretical accounts (10), or partially data-driven using factor analysis on predefined subscales that are based on *a priori* theoretical accounts (14, 22–24). Clearly, subjective categorization of TPQ results was not optimal given the low internal consistency at the subscale level and the poor goodness-of-fit (12, 15, 16, 19) On the other hand, given the binomial distribution of responses to TPQ questions/items (true/false), factor analysis of the TPQ data can lead to biased conclusions given its assumption on the Gaussianity of data (25, 26). Nevertheless, previous studies used factor analysis at the question/item level and could not replicate Cloninger’s TPQ structure (14, 22–24). In comparison, ICA does not assume data normality (70), as evident in TPQ data. Furthermore, ICA defines statistical independence using higher order moments as opposed to decorrelation with factor analysis (30). Application of ICA produced 13 independent components that represent the data-driven TPQ structure. ICA components were drastically different from the 12 subscales of Cloninger’s TPQ structure as well as the 3-factor solution of EFA (10, 12).

Our data-driven components exhibited consistently moderate to high test-retest reliability was across all components, thus confirming the reliability of the data-driven TPQ structure. However, the internal consistency was poor overall, with the exception of IC1, IC2, and IC7, which were deemed acceptable. This is likely due to the fact that the independent components represent statistically independent, and mostly nonlinear, latent variables with weights derived from all questions rather than from a subset of questions only. As such, ICA loadings did not uniquely relate to any specific subset of the TPQ questions (or Cloninger’s subscales or EFA factors). As such, our results confirmed the difference between data-driven personality components and the 12 TPQ subscales proposed by Cloninger (10, 12).

TPQ has been widely used to study psychopathology and psychotropic treatment effects. Here, however, we used TPQ metrics as potential diagnostic markers for MDD. We further compared the clinical utility of the data-driven vs. the psychological structure of the TPQ in identifying MDD in a sizeable sample of patients and healthy individuals. The data-driven components of TPQ were significantly more superior to the psychological structure of the TPQ in distinguishing medication-naïve patients with MDD from HC. Specifically, both accuracy (identification of MDD and HCs) and recall (identification of MDD) were more pronounced for the data-drive classifier. This might be attributed to the nonlinear and non-Gaussian examination the data compared to factor analysis, or factor-analysis-based categorization methods. These methods are often criticized as being simple, and unable to capture the complexity of underlying psychological processes (65). This result holds significant potential for data-driven reexamination of psychometric results to extract statistically independent components that link better to underlying behavioral and neural constructs or subsequent diagnosis and treatment choices (71).

Finally, we fully recognize that identifying statistically-independent components does not necessarily equate with measuring underlying biological processes of temperaments or personality characteristics, which are often more complex and interdependent (65). F (72) or instance, the functional ensemble of temperament (FET) model highlights the role neurotransmitter system in the regulation of temperaments, with their items reflecting the dynamics of behavior (31, 66, 72). Although data collection for our study predates the neurochemically-informed FET, examination of results from the FET model using our approach would shed a very important light on the correspondence between data-driven temperaments and their neurochemical basis. In fact, we argue that every study using psychometrics should cross-examine the data-driven and theory-based structure of their results to highlight latent variables that could link different levels of analysis.

To our knowledge, this is the first study that combines advanced analysis of TPQ structure, the utilization of machine learning classifiers to identify MDD, and a significant sample size of both patients and matched controls. In essence, this paper presents a roadmap for the reexamination of a huge body of literature on psychometrics. Immediate positive outcomes for the field of mental health can emerge in the form of improving clinical utility, replicability, and cross-discipline inferences.

## Data Availability

The raw data supporting the conclusions of this article will be made available by the authors, without undue reservation.
